# Quality Tuberculosis Care in Indonesia: Using Patient Pathway Analysis to Optimize Public–Private Collaboration

**DOI:** 10.1093/infdis/jix379

**Published:** 2017-11-06

**Authors:** Asik Surya, Budiarti Setyaningsih, Helmi Suryani Nasution, Cicilia Gita Parwati, Yullita E Yuzwar, Mike Osberg, Christy L Hanson, Aaron Hymoff, Pia Mingkwan, Julia Makayova, Agnes Gebhard, Wiendra Waworuntu

**Affiliations:** 1 Subdirectorate of Tuberculosis, Ministry of Health, Jakarta, and; 2 Challenge TB Project, KNCV Tuberculosis Foundation, Jakarta, Indonesia;; 3 Linksbridge, Seattle, Washington,; 4 Macalester College, St Paul, Minnesota, and; 5 Bill & Melinda Gates Foundation, Seattle, Washington; and; 6 Directorate of Prevention and Control of Communicable Disease, Ministry of Health, Jakarta, Indonesia

**Keywords:** tuberculosis, patient pathway analysis, care seeking, Indonesia, private sector

## Abstract

**Background:**

Tuberculosis (TB) is the fourth leading cause of death in Indonesia. In 2015, the World Health Organization estimated that nearly two-thirds of the TB patients in Indonesia had not been notified, and the status of their care remained unknown. As such, Indonesia is home to nearly 20% of the world’s “missing” TB patients. Understanding where patients go for care may enable strategic planning of services to better reach them.

**Methods:**

A patient pathway analysis (PPA) was conducted to assess the alignment between patient care seeking and the availability of TB diagnostic and treatment services at the national and subnational level in Indonesia.

**Results:**

The PPA results revealed that only 20% of patients encountered diagnostic capacity at the location where they first sought care. Most initial care seeking occurred in the private sector and case notification lagged behind diagnostic confirmation in the public sector.

**Conclusions:**

The PPA results emphasize the role that the private sector plays in TB patient care seeking and suggested a need for differentiated approaches, by province, to respond to variances in care-seeking patterns and the capacities of public and private providers.

In 2015, the 193 member states of the United Nations endorsed the Sustainable Development Goals (SDGs), reflecting broad aims to end poverty and improve the health and well-being of all people [1]. With the fourth largest population in the world, Indonesia’s progress toward the SDGs will be a key driver for global success [2]. Indonesia has made enormous gains in poverty reduction, with poverty rates declining by half since 1999, to 10.9% in 2016 [3]. However, >28 million Indonesians still live below the poverty line and approximately 40% of the population remains vulnerable to poverty, with incomes hovering marginally above the national poverty line [4].

Tuberculosis (TB), widely recognized as a disease of poverty, continues to threaten individual and national economic development in Indonesia. Tuberculosis is the third leading cause of death and fourth highest contributor to disability-adjusted life years (DALYs) in Indonesia [5]. A 2014–2015 TB prevalence survey estimated Indonesia’s burden at 1 million cases, placing the country second in the world for total TB burden [6]. Despite the success of the National Tuberculosis Control Program (NTP) in reaching key international indicators for treatment success (>80%), reporting of cases and early diagnosis remain a challenge [7]. In 2015, nearly two-thirds of the estimated new cases of TB were not notified and the status of these patients’ diagnosis and care is not known [7, 8]. The 689 271 missing cases represent approximately 19% of all missing cases worldwide [7].

Finding the missing TB patients and ensuring that they are cured, without incurring impoverishing costs, is a priority for the government of Indonesia. This priority is reflected in the national health sector strategy and national strategic plan for TB [4, 8]. Operationally, finding the missing cases will require knitting together a complex array of health actors across a vast territory to create a seamless care network. Spread across an archipelago of >17000 islands, Indonesia features a decentralized model of governance; its 34 provinces each have a governor and legislature [4]. Healthcare is similarly decentralized, with the main administrative and financial responsibilities residing at the district level. In 2012, the Ministry of Health began efforts to reinforce primary healthcare through the existing decentralized public care center network—that is, strengthening the capacities of facilities known as “Puskesmas” [4]. A referral system connects Puskesmas with district, provincial, and central hospitals, which provide secondary and tertiary care [4].

The private sector manages >50% of the hospitals in Indonesia [4, 9]. There are an estimated 70000 private practitioners [10]. Additionally, roughly 60%–70% of public employees have secondary employment in private health facilities or private practice [4]. Given this network, the private sector provides approximately 60% of outpatient care and 43% of hospital admissions. However, the private sector contributed only 9% to the notified TB cases in 2015 [10].

The End TB Strategy, promoted by the World Health Organization (WHO), is anchored in the importance of patient-centered care [11]. Finding and curing the missing TB patients in Indonesia requires a more robust understanding of how patients navigate the complex healthcare network, and how TB services can be best positioned to meet patients where they are. The aim of this study is to use existing population-based and health systems data to assess the alignment of care-seeking behavior and TB service delivery. The hope is that identifying systemic gaps to providing patient-centered care can result in more targeted strategic planning.

## METHODS

The patient pathway analysis (PPA) methodology described by Hanson et al [12] was used to assess the alignment between patient care seeking and the availability of TB diagnostic and treatment services. PPAs were completed at the national level and subnationally for 34 provinces. The primary data points considered by the PPA were initial care-seeking patterns for presumptive TB patients, availability of smear microscopy as a proxy for diagnostic availability, location of TB diagnostic confirmation, TB treatment initiation and notification, and documentation of treatment success. The data sources for each are shown in Table 1, and are described in more detail below. Further background on each data source is provided in the Supplementary Appendix to this article.

**Table 1.** Primary Data Sources

PPA Component	Data Source
Care seeking for TB symptoms	2014 National TB Prevalence Survey
TB diagnostic availability	2011 Risfaskes (Service Provision Assessment)
TB diagnostic location	2010 Riskesdas (Basic Health Survey)
TB treatment location – all cases (national only)	2014 National TB Prevalence Survey
TB treatment location – notified cases	SITT TB Surveillance Database (accessed 10 Jan 2017), 2016 WHO Global TB Report
Treatment success rate	SITT TB Surveillance Database (accessed 10 Jan 2017), 2016 WHO Global TB Report

Abbreviations: SITT, Sistem Informasi Tuberkulosis Terpadu; TB, tuberculosis; WHO, World Health Organization.

In some cases, several data sources provided similar data relevant to the PPA. In these cases, data sources that explicitly addressed TB patients or services were prioritized over general illness-related data. For example, both the National TB Prevalence Survey and National Susenas survey provided data on patient care seeking. Because the prevalence survey addressed care seeking among TB symptomatic patients whereas Susenas addressed patients with general illness, the prevalence survey was prioritized for the PPA. After TB-related data, data sources that were subnationally representative or more recent were prioritized. Additional details on prioritizing data sources for use in the PPA are provided elsewhere [12].

Each of the data sources used a different naming convention for health facilities. To allow for comparison across data sources, common categories were created to designate individual facilities as being public, private (formal), or private (informal), and to classify them as 1 of 3 levels in the health system. Specifically, the following facility categories were used:

• Level 0 (L0): Refers to the most basic and usually community-based care level. Level 0 services include basic triage, health information, and essential prevention and care. Services are commonly provided as an extension of facility-based care, and are provided by volunteers or paramedical staff with limited formal training. No laboratory testing is available but L0 staff may serve as treatment supporters for TB patients. Examples: village health posts for maternal and child health care and clinics for the elderly, UPAYA KESEHATAN BERBASIS MASYARAKAT (public), and pharmacies, drug sellers (private), community based organizations (with health cadres), dengue teams.
• Level 1 (L1): Refers to a facility that provides primary health care. Nurses, midwives, or private doctors commonly provide L1 services, generally on an outpatient basis. Some basic diagnostic services and essential medicines may be available. Examples: Puskesmas/health center (public); and private clinic (private).
• Level 2 (L2): Refers to facilities that provide primary health care as well as more advanced care. L2 facilities commonly have more extensive diagnostic and treatment options and can provide both outpatient and inpatient care. Examples: any public hospital (public) and nongovernmental organization or private hospital (private).
• Level 3 (L3): Refers to facilities that provide specialized care with large inpatient capacity. L3 facilities provide access to specialized doctors and have more sophisticated diagnostic and treatment capabilities. Example: tertiary care hospitals.

Table 2 provides a detailed mapping of the health facilities from each data source to the standard categories described above.

**Table 2.** Health Facility Mapping

Data Source	Facility Type (From Survey)	Mapped to →	Facility Sector	Facility Level
Prevalence Survey 2014	Other		Other	Other
	Private-sector hospital		Private sector	Level 2
	Nurse or midwife		Private sector	Level 1
	Private clinic		Private sector	Level 1
	Private practitioner		Private sector	Level 1
	Pharmacy/drug shop		Private sector	Level 0
	Public sector hospital		Public sector	Level 2
	Primary health facility		Public sector	Level 1
Rifaskes 2011	Private hospitals		Private sector	Level 2
	Practitioner doctor center		Private sector	Level 1
	Private practitioners		Private sector	Level 1
	Public hospitals (type C and D)		Public sector	Level 2
	Public hospitals (type A and B)		Public sector	Level 3
	Puskesmas		Public sector	Level 1
	UKBM		Public sector	Level 0
Riskesdas 2010	Not treated		Not treated	Not treated
	Private sector hospital		Private sector	Level 2
	Clinic/private practitioner		Private sector	Level 1
	Public sector hospital		Public sector	Level 2
	Primary health facility		Public sector	Level 1

Abbreviations: UKBM, UPAYA KESEHATAN BERSUMBERDAYA MASYARAKAT (Bersumberdaya Public Health Efforts).

**Figure 1.**
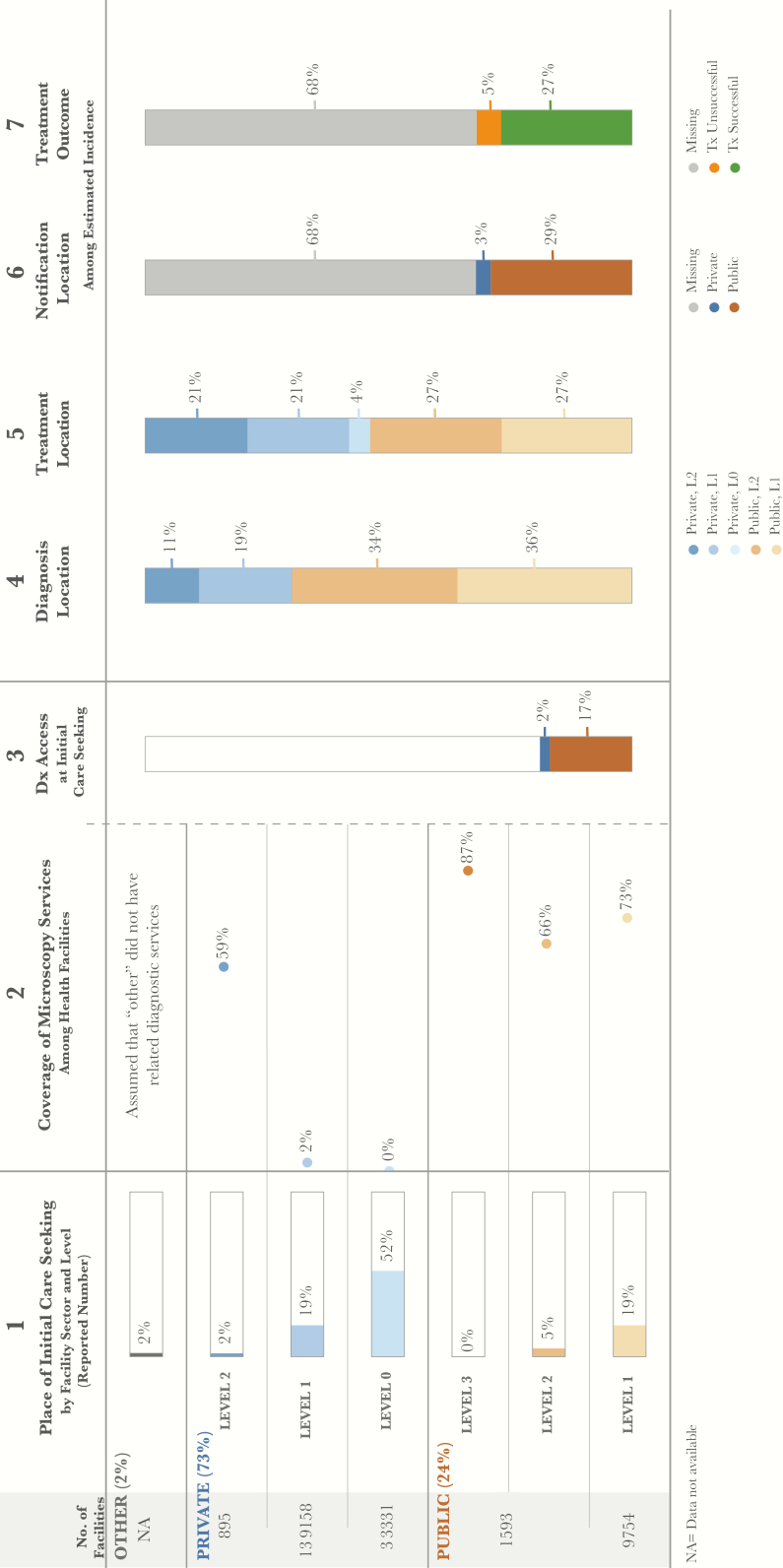
Patient pathway visual, national level. The patient pathway describes the care-seeking patterns of patients and how those patients may intersect with tuberculosis (TB) services. Column 1 starts by showing the sectors and levels of the health system (sectors and levels where no data was available are not included in the pathway, eg, public L0). The percentage next to each sector title is the share of patients who initiate care seeking in this sector [6]. Next is the estimated number of health facilities at each level within each sector [10]. The final part of column 1 shows the location of initial care seeking among participants in the 2014 National TB Prevalence Survey at which patients sought care for TB symptoms (14 days of cough or hemoptysis) at each level of the health system [6]. Column 2 shows the percentage of health facilities that have microscopy across each sector and level of the health system (ie, coverage) [10, 14]. Column 3 shows the estimated percentage of patients likely to access a facility with TB diagnosis available on their initial visit to a healthcare facility. This column was calculated by multiplying the share of care seeking at each sector/level of the health system by the coverage of microscopy at each respective sector/level and summing the total. Column 3 separates public and private sectors based on each sector’s contribution to TB services access at initial care seeking. Column 4 shows the TB diagnosis location of participants in a 2010 national-level health survey [15]. Column 5 shows the location of treatment among those participants who were diagnosed with TB in the 2014 National TB Prevalence Survey [6]. Column 6 shows which sector provided case notification and is calculated as a share of the overall estimated incidence in 2015 [7, 13]. Column 7 shows the treatment outcome of notified cases among the overall estimated incidence for 2015 [7, 13]. Columns may not add to 100%, due to rounding. For more details on the data sources used in the pathway, see the Supplementary Materials. Abbreviations: Dx, diagnosis; Tx, treatment.

The number of health facilities at each level is included for public and private facilities at the start of the pathway [10]. Estimates of initial care seeking were derived from the 2013–2014 national TB prevalence survey [6]. Among participants (N = 67944) with TB symptoms (n = 8552), an indicator captured participants who sought care for those symptoms (n = 4867). These estimates are shown as column 1 in the patient pathway visual (Figure 1). It is important to note that L0 care seeking in the public sector was not recorded as a care-seeking option in the prevalence survey. There are several L0 public sector facilities that are involved in TB care, namely Posyandu, Pos TB Desa (village TB post), and Posbindu. Each of these facilities may play a role in either finding and referring TB cases or supporting patients who are on treatment. They are community-level providers and many of them throughout the country are supported by civil society organizations and supervised and coordinated by Puskesmas at L1. Very few data were available about the activities of L0 facilities, but SITT did record that 0.6% (n = 1928) of notified TB cases were referred from these facilities in 2015 [13].

Rifaskes, a national-level service availability survey, was completed in 2011 and provided information on the availability of smear microscopy for TB diagnosis [14]. At the provincial level, these coverage data were only available for a census of public sector L1 (n = 8981 Puskesmas) and a survey of L2 facilities (n = 685 hospitals). At the national level, the survey also estimated microscopy availability within a sample of L2 private-sector facilities (n = 30). These national-level data of private sector L2 coverage were extrapolated to all provinces to enable an estimate in each of the provincial level PPAs. Rifaskes reported that among L3 hospitals, hospitals classified as type A all had microscopy, while just over 85% of type B hospitals had microscopy.

For private-sector level 1 facilities, an estimate was provided as part of the country program update prior to the 2017 Joint External Monitoring Mission that suggested that 2% of private sector facilities were covered by DOTS services, including diagnosis [10]. This figure was used as the L1 private sector estimate for each of the provinces as well as the national PPA. The true coverage levels for both L1, L2, and L3 are unknown, but likely to vary in different provincial contexts. Because of this, the PPA may underestimate coverage in some provinces and overestimate it in others. Furthermore, as highlighted in the introduction, many general practitioners in Indonesia work in both the private and public sectors. The extent of this overlap is unknown, but should be considered when interpreting these figures. These private sector coverage estimates are shown as column 2 in the patient pathway visual (Figure 1).

To estimate the likelihood of a TB patient accessing diagnostic services at the point-of-care initiation, the percentage of patients who sought care at each health sector or level was multiplied by the coverage of TB diagnostic services in that category. This calculation was made for each health facility category. The results are summed to provide an estimate of the accessibility of TB diagnostics at first care visit. These estimates are shown as column 3 in the patient pathway visual (Figure 1).

Data were not available to estimate the pattern of care seeking beyond the initiation of care. However, the location of TB diagnostic confirmation was available from the 2010 basic health survey, Riskesdas [15]. These data are reflected in column 4 of the patient pathway visual (Figure 1).

Columns 5 and 6 (Figure 1) show 2 alternatives to estimating the location of treatment for confirmed TB patients. Column 5 reflects the estimates derived from the national TB prevalence survey [6]. Specifically, the data captured the location of current treatment for all TB patients identified during the prevalence survey. SITT, the national TB surveillance system, reports the location of treatment for notified TB patients. In 2015, notified cases were primarily being treated in the public sector, which accounts for 91% of notified cases, whereas the private sector only notified 9% of cases [13]. However, a 2011 study suggests that 62% of TB treatment occurred in the public sector and 38% occurred in the private sector [16], indicating that the low notification rate in the private sector leads to an underestimation of TB treatment in private facilities. Column 6 (Figure 1) reflects the location of treatment for TB patients notified through SITT. The data in this column show the notified cases calculated against a denominator of total estimated incident cases in 2015, when notified cases covered 32% of the estimated burden.

The treatment success rate reported to the NTP is recorded in column 7 (Figure 1), but was applied only to notified patients. The treatment success is reported in the pathway as a fraction of the total estimated burden, which also shows those cases that were not notified (ie, missing). The resulting PPA (Figure 1) estimates the steps a patient may take on the pathway to accessing TB care.

There are some important limitations to the methodology described above. First, these data are not based on a single cohort, but rather several aggregate data sources in an attempt to approximate what the path of a cohort might look like. The analysis assumed that the patterns among these time periods were relatively constant though many of the data sources are from different time periods; this may not reflect reality. Patient pathways were created for 33 provinces, which emphasized important subnational differences in care-seeking patterns and service availability. The prevalence survey, which provided the data on care seeking among symptomatic patients (n = 4867), was not powered to the subnational level. Therefore, there is a level of uncertainty among the subnational patterns described below. Furthermore, there were few data on the coverage of microscopy among private sector providers. Given the importance of these providers in caring for TB patients, this is an important area for future research to ensure that patients seeking care in the private sector are appropriately cared for. Further limitations of the PPA methodology are described elsewhere [12].

## RESULTS

### The Majority of Initial Care Seeking Occurred in the Private Sector

Nearly three-quarters of patients sought care for TB symptoms in the private sector. Given the paucity of subnational data on diagnostic capacity in the private sector, the efficiency of diagnosis in this sector cannot be well described. However, more than half of patients seeking care in the private sector visited level 0 facilities, such as drug shops and pharmacies, where diagnostic confirmation would not be expected to be available.

### Less Than 20% of Patients Encountered Diagnostic Capacity Where They Initiated Care

**Figure 2.**
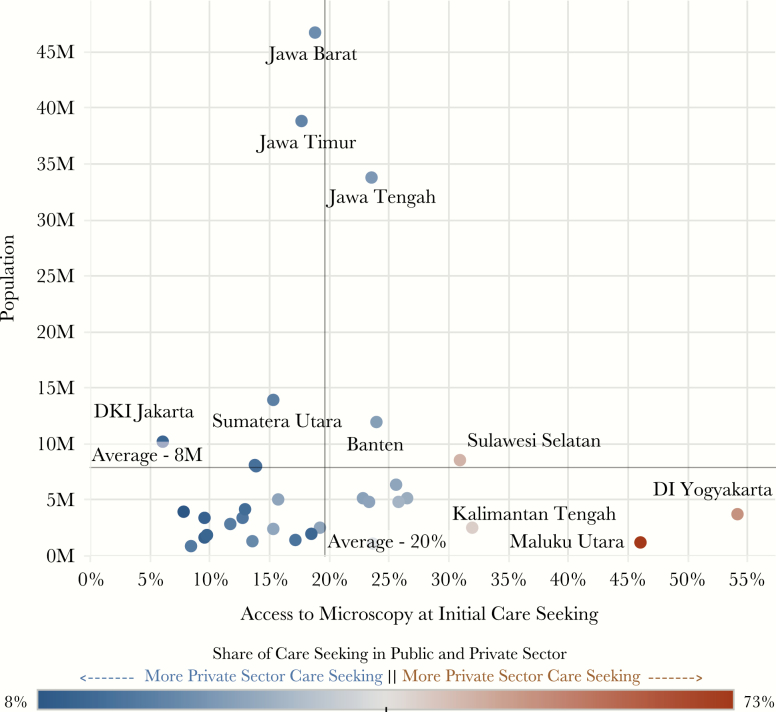
Access to microscopy at initial care seeking by province. The patient pathway analysis was completed for 33 of 34 provinces in Indonesia. This figure shows the access to diagnosis at initial care seeking for each province (column 3 of the patient pathway visual) as well as the population of each province. Dots representing each province are colored based on the share of initial care seeking in public (brown) vs private (blue) health facilities [6]. Abbreviations: DI, Daerah Istimewa; DKI, Daerah Khusus Ibukota.

At a national level, only 19% of TB patients accessed a facility known to have smear microscopy services as their first point of care (Figure 1, column 3). There were important provincial-level differences in access to diagnosis at the time of initial care seeking (Figure 2). Considering that 73% of L1 and L2 public health facilities had smear microscopy available, provinces with high care seeking in the public sector had a greater percentage of patients seeking care at a facility with diagnostic capability (eg, Yogyakarta at 53% and Maluku Utara at 46%). Conversely, provinces with high care seeking in the private sector typically had a relatively small percentage of patients seeking care at a location with smear microscopy (eg, Papua Baratat at 8%, Kalimantan Selatan at 8%, and DKI Jakarta at 6%). In 60% of provinces (n = 20), between 10% and 19% of patients initiated care in facilities with smear microscopy. In 9 provinces, this rate was between 20% and 30% and in only 4 provinces was it above 30%.

Figure 1 (column 4) suggests that patients who initiated care with L0 private providers had to transition to the public sector or higher-level private providers for diagnosis. In the public sector, a greater proportion of confirmed TB patients (70%) reported having received a diagnosis in the public sector than initiated care in this sector (24%, column 1). This shift was particularly notable at higher levels of the public sector; for example, whereas 5% of patients initiated care in L2 public facilities (column 1), 35% of confirmed TB patients reported being diagnosed there (column 4).

### Case Notifications Were Lower Than Diagnostic Confirmations, Even in the Public Sector

Initial care-seeking behavior was considered a proxy for patient preference given the perceived affordability and appropriateness that enabled access. After diagnostic confirmation, which appeared to not commonly take place at the site of care initiation, the findings suggest that patients sought treatment with providers who corresponded more closely with their care preferences. Specifically, it appears that among patients with diagnostic confirmation for TB, 70% received their diagnosis in the public sector (Figure 1, column 4), whereas only 54% of patients who were started on treatment (column 5) did so in the public sector. Thus, it appears that patients may transition back to the private sector for treatment after receiving diagnosis in the public sector.

Acknowledging the potential confounders of multiple data sources, the pathway suggests a storyline of the patient experience. While only 24% of initial care seeking was in the public sector, 70% of confirmed TB patients were diagnosed there (columns 1 and 4, respectively). Then, 54% of treatment was reported to take place in the public sector but only 28% of all estimated cases were notified by the public sector (columns 5 and 6, respectively). Given this, it appears that either patients were lost between diagnostic confirmation and treatment initiation or not all patients initiated on treatment were notified through the NTP’s recording and reporting system. A case-based electronic data capture system was introduced in 2014 and reached full coverage in 2015. However, there remain challenges with data entry given limited internet access. There is not yet a corresponding electronic system for laboratory-based data. While mandatory reporting of TB was introduced in 2017, the electronic reporting system has primarily been used by the public sector to date. While data capture may explain some of the apparent drop-off in patients, a recent epidemiological review suggested a loss of patients between treatment initiation and treatment completion in the public sector. Undernotification among diagnosed cases was also a key finding in the 2013–2014 prevalence survey [6].

At the subnational level, the location of initial care seeking compared to the level of healthcare facility where treatment occurred revealed misalignment in many provinces. The findings suggest that patients moved to higher-level facilities and possibly between sectors for diagnostic confirmation and treatment. The provinces of Maluku Utara and DI Yogayakarta, for example, achieved patient notification rates in the private sector that were nearly commensurate with the proportion of initial care seeking in the private sector. However, the level at which care was obtained varied across the continuum of care. Based on aggregated data, patients were tracked back to their inital care-seeking preferences, which showed that while only 10% of patients in Yogyakarta initially sought care at an L2 private facility (ie, hospital), >32% of treatment (among notified cases) took place at this level. Similarly, nearly all initial care seeking in the private sector in the province of Maluku Utara took place at L0 (ie, pharmacies and drugstores). However, all notified treatment in the private sector was reported from the L1 and L2 levels.

Nationally, the share of TB case notifications from the private sector was 65 percentage points lower than the percentage estimated to initiate care seeking in the private sector. There was wide variance in this gap across provinces. In 2 provinces, Yogyakarta and Maluku Utara, the gap was <15 percentage points, suggesting private sector engagement in the care of TB patients that was nearly commensurate with their estimated patient load. In Yogyakarta, approximately half of care seeking was initiated in the private sector. In all but 5 provinces, there was a >50 percentage point difference in the proportion of patients seeking care in the private sector compared with the proportion of TB patients notified. In two-thirds of provinces, at least 75% of initial care seeking was estimated to take place in the private sector. Comparing the share of care seeking in the private sector to the share of notified cases from the private sector reiterates the finding from the 2013–2014 prevalence survey that many private sector facilities likely underreport cases to the NTP. These notified cases likely only capture a fraction of the cases that are actually cared for in the private sector, and provinces with high care seeking in the private sector may provide a starting point from which missing cases could be located.

## DISCUSSION

The patient pathway analysis was the first exploration at a national and subnational level of the nature of the misalignment between the availability of TB services and patient care-seeking behaviors. The study provided a systematic comparison across provinces of the relative importance of the different sectors and service delivery levels for TB case finding and treatment. The results provide a framework for shifting programmatic priorities to better meet patients where they are. They also highlight where further operational research may be needed to understand which solutions to overcoming gaps will be most effective.

Many previous studies and programmatic data have highlighted the important role of the private sector in Indonesia [17–20]. The PPA further demonstrated the complex interplay between the public and private sectors when it comes to the management of TB patients. However, the paucity of data from the private sector made it difficult to fully describe the patient experience in the private sector. Attaining better data from the private sector will be essential for evidence-based planning of patient-centered interventions. The 2017 introduction of mandatory reporting is a welcome policy foundation that may promote more consistent reporting from private sector providers. However, the uptake of this policy will require interventions to enable and enforce its use.

### Universal Health Coverage May Bring Efficiencies to Public–Private Collaboration

Since 2014, Indonesia has implemented universal health coverage through the National health insurance provider, Badan Penyelenggara Jaminan Sosial (BPJS) Kesehatan [4, 9, 21, 22]. Currently, BPJS Kesehatan covers almost 70% of the total population and includes almost all public health providers and the majority of private hospitals [4, 21, 22]. However, inclusion of private general practitioners and specialists is still very limited. There are an estimated 120000–130000 private practitioners in Indonesia, with the number engaged in BPJS limited to <10000 (approximately 8%) [10, 23]. TB services are guaranteed by BPJS Kesehatan through tiered referrals. Official regulations require that only complicated TB cases be referred to L2 facilities, as L1 public and private health facilities should be able to manage uncomplicated TB cases; but ensuring compliance with this regulation has been challenging.

Due to the limited availability of microscopes and other diagnostic tools at L1 private facilities, there are often diagnostic referrals to L1 public facilities or even L2 public and private facilities. According to BPJS Kesehatan regulations, after receiving diagnostic results, patients with uncomplicated TB should be referred back to the L1 referring facilities [22]. However, some uncomplicated cases remain in the L2 facilities. This lack of regulatory compliance results in diagnostic delays, higher costs for patients, and unnecessary expenditures from BPJS Kesehatan because the cost of uncomplicated TB case at L2 is higher. Improving compliance with regulations would require a change in behavior for both patients and primary care providers to ensure that patients with TB symptoms are screened for TB at the primary care level prior to referral to secondary levels.

Improvements in the health insurance reimbursement structure, alongside targeted training of private L1 providers, may be needed to stimulate appropriate referrals from these providers. Given the movement of patients between levels and sectors, the NTP, BPJS Kesehatan, professional organizations, health provider associations, and other relevant stakeholders will need to collaborate to introduce a more rational and efficient referral system as part of addressing the gaps highlighted by the PPA. Additionally, raising community awareness about service availability and TB symptoms may influence demand and result in more efficient care seeking.

### A Case for Strengthening Public Sector Capacity

The important role of the private sector in TB care has been acknowledged. However, the role of the public sector should not be forgotten. An estimated 77% of TB patients who initiated care in the public sector accessed facilities that had at least sputum smear microscopy. However, not all Puskesmas have the capacity to provide basic microscopic examination. Closing this gap should be considered low-hanging fruit. Puskesmas without laboratory capacity generally send a fixed sputum smear slide, rather than the patient, to a microscopic referral site in another Puskesmas. The microscopic referral site will send back the result to the referring Puskesmas. Patients with positive results start the treatment in the referring Puskesmas. In an effort to reduce delays, the country aims to ensure microscope availability in all Puskesmas by 2020. The expansion of new technologies is also being planned and can be prioritized based on the findings of this PPA.

Comparing the coverage of smear microscopy across health facility levels to the share of patients who sought care at each level, the PPA highlighted differences in the structure of diagnostic systems within each province. Some provinces had higher coverage of diagnostic services available where patients sought care (eg, Yogyakarta and Maluku Utara), whereas others were more reliant on centralized diagnostic centers with referral or sputum transport networks connecting patients to these providers (eg, Jakarta). Unfortunately, there were insufficient data available to determine whether low diagnostic coverage in a province was the result of an intentional structure of centralized diagnostics or resource constraints that led to insufficient diagnostic capacity. Within each context, the provincial PPAs can be interpreted to determine where diagnostic coverage, referral, and sputum transport systems need to be improved and where patients might be at greatest risk for diagnostic delay. This is likely to be particularly important where private-sector care seeking is high and diagnostic coverage in the private sector appears to be low.

### Quality of Services may be as Important as the Coverage of Services

While the PPA demonstrated gaps in technology and commodity availability, it did not measure the capacity to implement or the quality of service provision. According to the 2011 Rifaskes survey, 89% of L1 Puskesmas had a microscope available. However, only 73% had the trained human resource capacity and commodities available to complete a test. At the hospital level, nearly all public (97%) and private (100%) hospitals had a microscope available, though only 73% of public and 59% of private hospitals had the capacity to provide a TB test.

A study by Probandari et al found that even in public–private hospitals, 19%–53% of providers did not follow standardized diagnosis and treatment protocols [17]. Another study conducted in 8 major cities in Indonesia found that the TB diagnostic and treatment practices among private general practitioners (L1) did not meet the International Standards for Tuberculosis Care [18]. The authors suggested that the capacity and authorities of the public sector were insufficient to steward or enforce compliance by private providers to government standards. However, some case studies that offer lessons for replication are emerging. A study in Mimika, Papua, for example, found that collaboration—including training, supervision, and quality assurance of laboratory services—involving the district health office, nongovernmental organizations, and private companies resulted in treatment success rates close to 90% [19]. As data emerge from the private sector, both through mandatory reporting and through health insurance claims, further analysis can inform quality improvements in this sector.

### Role of Pharmacies Cannot be Overlooked

The 2014 Prevalence Survey quantified the important role of the private sector as a provider of TB care in Indonesia. The results of the PPA showed that nationally, high levels of initial care seeking (52%) occurred in L0 private-sector sites, with variation at the provincial level ranging from 17% in Yogyakarta to 74% in Maluku. The use of L0 may be due to easy access to drugs in pharmacies and drug shops. Indonesian drug regulations mandate that only over-the-counter or nonprescription drugs be sold by a pharmacy directly to end customers [4]. TB drugs, which are classified as prescription-only medicines, can be readily obtained without prescription in many pharmacies and drug shops. The widespread availability of unregulated anti-TB medicines in the private sector has been documented in other studies [4, 16]. Several actions have been proposed and initiated at the national level by the NTP and technical partners. The results of the PPA suggest a prioritized approach in those provinces with considerable care seeking at L0 and limited capacity for oversight by, or alternative treatment options within, the public sector. Determining how to best incentivize, regulate, and support pharmacy engagement in TB care through the NTP’s collaboration with the pharmacy association will be essential for increasing case finding and preventing TB drug resistance. In addition, stronger drug regulation enforcement should be introduced to ensure that pharmacies and drug shops adhere to existing drug regulations.

Although TB drugs are free through public facilities in Indonesia, unless there is collaboration between public and private facilities, TB patients treated at private facilities have to spend out-of-pocket money for their treatment. Collaboration between the public and private sectors, therefore, is very important in areas where the presence of private facilities is high. Patients should always be able to access free TB drugs regardless of their treatment location.

Raising community awareness could also influence initial care-seeking behavior and the referral behavior of initial care providers, including pharmacies. Presumptive cases that are referred by community members to Puskesmas and are diagnosed and recorded as confirmed TB are tracked as “community referral” cases. Community referrals are an important target indicator in the strategic plan and could help to reduce gaps in the patient pathway, especially if focused on identified at-risk groups.

The prevalence survey suggested that nearly 43% of bacteriologically confirmed TB cases were among people who did not report symptoms. Further research is needed to fully understand the implications of these findings on care seeking (eg, the progression of symptoms over time). Pharmacies may play an important role in the early detection of presumptive TB, as symptoms emerge.

### Patient Pathway Analysis can Inform the Scale-up of the Xpert Assay and Multidrug-Resistant TB Care

Understanding the contribution of private facilities along the patient pathway also provides direction on where the Xpert assay should be placed for future expansion; that is, in provinces where private facilities play a prominent role, placement of Xpert should include the private facilities, after taking into account their available structure and staffing. Moreover, understanding where most cases are diagnosed (L1/L2) should direct whether Xpert or other diagnostic tools are placed in primary or secondary health facilities.

The knowledge provided by the PPA, from location of initial care seeking to diagnosis and treatment location, is important for the expansion of multidrug-resistant (MDR) TB care. Expanding MDR-TB care without addressing the need for increased treatment success could result in the creation of extensively drug-resistant TB cases, particularly in areas with a high proportion of private facilities.

A study by van Kampen et al demonstrated an important increase in the initiation of drug-resistant TB treatment and reduced diagnostic and treatment delays when the Xpert MTB/RIF assay was used in the diagnosis and treatment of presumptive drug-resistant TB patients in Indonesia [24].

The availability of rapid molecular testing is expanding in Indonesia. With this in mind, it may be valuable to repeat the PPA in the coming years once rapid molecular testing is more widely available. A repeat PPA could be beneficial in evaluating patients’ access to this new diagnostic tool and to evaluate its efficacy in increasing early diagnosis of MDR-TB and reducing treatment delays.

## CONCLUSIONS

The PPAs provided an evidence base from which to consider how to strengthen patient-centered care, make services available where patients are, and target services to different care-seeking behaviors in different parts of the country

Availability of information from the PPA, alongside an epidemiological review, enabled consideration of province-specific data as an input to the Joint External Monitoring Mission in January 2017 in Indonesia.

An important limitation of this PPA is the paucity and incompleteness of individual longitudinal patient data and a reliable cohort analysis regarding access to care as well as the lack of information on patients whose information was not captured, as they were not diagnosed and/or notified. The health network mapping in combination with patient and provider interviews at all steps of the patient pathway would be useful to improve the strength of the conclusions and their applicability for planning and modeling of interventions.

If based on sufficiently high-quality data, PPAs may provide a valuable lens through which country and district-level TB control officers can view decisions regarding strategies and approaches to combating TB.

In summary, this review emphasizes the needs of regulation, strong networking, and collaborations between various stakeholders to ensure collaboration between private and public health facilities. Engagement of stakeholders, such as NTP, province/district health offices, health insurance, professional organizations, patient and community groups, other ministries, partners, and other relevant actors, is mandatory.

TB notification is now mandatory by law, which reinforces the importance of strong enforcement. The PPA’s findings reiterate that the private sector plays a crucial role in engaging patients along their journey for care. It is crucial that the private sector report these cases in order for the NTP to reduce the number of missing cases in the country.

While the MOH and the NTP provide regulations, strategies, and guidelines, the implementation of TB control is mainly done at district and subdistrict levels, closely supervised and monitored by province/district health offices and professional organizations. Besides their role in community awareness and care, patient and community groups can act as a public watchdog, ensuring that the management of TB at local levels follows a patient-centered approach.

These strong, evidence-based networks and collaborations, together with regulations and appropriate technologies, are expected to lead to increased notification of TB and MDR-TB.

## Supplementary Data

Supplementary materials are available at *The Journal of Infectious Diseases* online. Consisting of data provided by the authors to benefit the reader, the posted materials are not copyedited and are the sole responsibility of the authors, so questions or comments should be addressed to the corresponding author.

## Supplementary Material

Supplementary Appendix

## Notes

***Supplement sponsorship.*** This work is part of a supplement sponsored by the Bill & Melinda Gates Foundation.

***Potential conflicts of interest.*** All authors: No reported conflicts of interest. All authors have submitted the ICMJE Form for Disclosure of Potential Conflicts of Interest. Conflicts that the editors consider relevant to the content of the manuscript have been disclosed.
