# Supplementary material for: Quality Tuberculosis Care in Indonesia: Using Patient Pathway Analysis to Optimize Public–Private Collaboration
Source: J Infect Dis. 2017 Nov 6;216(Suppl 7):S724–32. doi: 10.1093/infdis/jix379 (PMC5853837; doi:10.1093/infdis/jix379)
Supplement: Supplementary Appendix [file jix379_suppl_supplementary_appendix.docx]

| **Country: Indonesia** | | | | | |
| --- | --- | --- | --- | --- | --- |
| **Data Source** | **Survey Type** | **Survey Question** | **Reported Metric** | **Sample Size** | **PPA Step** |
| TB CONTROL PROGRAM UPDATE -  December 2016 (Provided prior to JEMM in January 2017) | N/A | N/A | Number of health facilities across public and private, including hospitals, general practitioners and primary care facilities (puskesmas) | N/A | Number of health facilities |
| 2014 National TB Prevalence Survey | Nationally representative survey of individuals aged 15 years and above | Did you look for medical treatment for this coughing episode? If yes, where did you go for the first time to seek medical treatment for this coughing episode? | Treatment-seeking pattern of participants who reported cough for 14 days or more or haemoptysis  (Table 15) | 67, 944 participants were included in the prevalence survey.  8,552 participants responded to question about whether (and where) they sought care for TB symptoms.  4,867 participants sought care for TB symptoms. | Step 1 –  Initial care seeking patterns |
|  |  | N/A | Distribution of participants who reported being under TB treatment by place of treatment (Table 35) | 67, 944 participants were included in the prevalence survey.  125 participants were currently under TB treatment and reported the location of current treatment. | Step 5 – Treatment location |
| 2011 Risfaskes (Service Provision Assessment)  Report Link: <http://labdata.litbang.depkes.go.id/riset-badan-litbangkes/menu-riskesnas/menu-rifaskes/149-rifas-2011> | National and regional public health facility based census whose aims were to evaluate the adequacy and appropriateness of health facility services | Tuberculosis diagnostic services through microscopic examination of TB is available? Yes/No | Percent of public hospitals with availability of microscopy for TB (Table 4.38)  Percent of puskesmas with sputum examination lab service  (Table 4.8.20.1.c) | From Supply-Side Readiness Analysis – “Rifaskes is census of 8,981 puskesmas and 685 public sector hospitals  Sample of 30 private sector hospitals”  (aiphss.org/wp-content/uploads/2015/02/Supply-Side-Readiness.pdf) | Step 2 – Coverage of microscopy among health facilities |
| 2010 Riskesdas (Basic Health Survey)  Report Link: <http://labmandat.litbang.depkes.go.id/riset-badan-litbangkes/menu-riskesnas/menu-riskesdas/148-rkd-2010> | Nationally representative survey | Have you been diagnosed with TB through sputum examination and/or chest Xray by health worker (doctors, nurses, midwives) in the past 12 months? If so, what is the location of the lab or facility that conducted the test (not the provider who requested it)? | Percent of type of health facility used for diagnosis of disease by people with TB >15 years old in the last 12 months (Table 3.4.3.14) | Sample representing the national level, 33 provinces and 441 districts/cities out of a total of 497 districts/cities in Indonesia. | Step 4 – Diagnosis location |
| SITT TB Surveillance Database (accessed 10/01/17) | National TB Registry | Download from SITT provided location of notification for each province and at the national level. Separated notification location by public, private and Level 1 and Level 2 facilities. | Percent of notified TB treatment location | N/A | Step 6 –  TB Treatment Location – Notified Cases |
| 2016 WHO Global TB Report | Annual report providing data on TB epidemiology, health systems and financing for 194 member state.  Notification location data available in table 4.2 of annual report.  Raw data accessed via Global TB Database available here:  <http://who.int/tb/data/en/> | N/A | Estimated burden – 1,020,000 cases  Treatment success rate – 84% | | Step 7 –  Among estimated burden-successfully treated cases |
